# Chemical modification of L-glutamine to alpha-amino glutarimide on autoclaving facilitates *Agrobacterium *infection of host and non-host plants: A new use of a known compound

**DOI:** 10.1186/1472-6769-11-1

**Published:** 2011-05-31

**Authors:** Indra Sandal, Amita Bhattacharya, Uksha Saini, Devinder Kaur, Shveta Sharma, Ashu Gulati, Jonnala K Kumar, Neeraj Kumar, Jyotsna Dayma, Pralay Das, Bikram Singh, Paramvir S Ahuja

**Affiliations:** 1 Center for Molecular Medicine and Infectious Diseases, Department of Biomedical Sciences and Pathobiology, Virginia Tech, Blacksburg, VA-24061, USA; 2 Department of Biotechnology, College of Life Sciences, Bowling Green State University, Bowling Green, Ohio, USA; 3 Central Institute of Medicinal and Aromatic Plants, Resource Centre, Boduppal, Hyderabad 500039 (A.P.), India; 4 CSIR-Institute of Himalayan Bioresource Technology, Council of Scientific and Industrial Research, Palampur-176061, H. P. India

## Abstract

**Background:**

Accidental autoclaving of L-glutamine was found to facilitate the *Agrobacterium *infection of a non host plant like tea in an earlier study. In the present communication, we elucidate the structural changes in L-glutamine due to autoclaving and also confirm the role of heat transformed L-glutamine in *Agrobacterium *mediated genetic transformation of host/non host plants.

**Results:**

When autoclaved at 121°C and 15 psi for 20 or 40 min, L-glutamine was structurally modified into 5-oxo proline and 3-amino glutarimide (α-amino glutarimide), respectively. Of the two autoclaved products, only α-amino glutarimide facilitated *Agrobacterium *infection of a number of resistant to susceptible plants. However, the compound did not have any *vir *gene inducing property.

**Conclusions:**

We report a one pot autoclave process for the synthesis of 5-oxo proline and α-amino glutarimide from L-glutamine. Xenobiotic detoxifying property of α-amino glutarimide is also proposed.

## Background

Glutarimides are the hydrolyzed cyclic imides formed as a result of cyclization of glutamine. These compounds particularly, the α-amino glutarimides are important components of many extremely valuable drugs. The glutarimide moiety is present in a great number of molecules and has a broad spectrum of pharmacological activities. Many glutarimides have been reported to show notable anticancer activity, cytotoxicity against KB cells *in vitro*, and are also potent inhibitors of P388 murine leukemia *in vivo *[1]. Since aminoglutethimide inhibits steroidogenesis at the aromatase sites, it is used in treating metastatic breast cancer [2]. Antineoplaston A10 (N-[(3S)-2,6-dioxo-3-piperidyl]-2-phenyl-acetamide) is another glutarimide derivative, originally isolated from human urine. It has remarkable anticancer activity but lacks the toxicity of other common cancer drugs [3]. It has been suggested that A10 acts directly at the genomic level and alters the cellular responsiveness to steroidal hormones [4]. Thalidomides have special clinical value in a number of pathological conditions such as erythema nodosum leprosum, rheumatoid arthritis, HIV-associated oral ulcers, and chronic graft *versus *host diseases [5]. Both *in vitro *and *in vivo *studies have shown that thalidomides have the ability to inhibit tumor necrosis factor-α and its production [6]. FDA has approved this drug for the treatment of erythema nodosum leprosum (ENL) and multiple myeloma [7]. Many glutarimide derivatives are partial agonists of the central nervous system (CNS) and their actions range from convulsive and analeptic (agonist) to anticonvulsive and hypnotic (antagonist). The importance of glutarimide moeties in antibiotics such as the cycloheximides is well known and they act by inhibiting the synthesis of bacterial peptides at the initiation and extension steps [8]. However, the present study elucidates and reports a new function of α-amino glutarimide as a potent facilitator of *Agrobacterium *infection of generally resistant host plants. The study also shows that α-amino glutarimide is a major product formed as a result of chemical modification of L-glutamine upon autoclaving.

Infection of target host and non host plants by engineered *Agrobacterium tumefaciens *is required for the production of tailor made transgenic plants expressing useful yield and quality enhancing genes [9]. As compared to direct transformation methods, *Agrobacterium *mediated genetic transformation allows higher frequency of stable transgene expression [10,11]. Hence this method has been employed to produce a large number of transgenic plants till date. Even the natural barriers that prevent the use of *Agrobacterium *as a vector for genetic transformation of monocots have now been overcome through the use of several innovative approaches, and several crop plants have been successfully transformed [11-16]. Yet, quite a good number of crops, particularly, several important ones that exude bacteriostatic or bactericidal leachates as plant defense have remained outside *Agrobacterium*'s host range, and gene transfer into them is largely difficult. It is while targeting such 'difficult to transform crops' for transgenic production by *Agrobacaterium tumefaciens*, that α-amino glutarimide can prove extremely useful.

## Methods

Aqueous solution of L-glutamine at a concentration of 2.0 g litre^-1 ^(Sigma, India) was autoclaved at 121°C and 15 psi for 20 and 40 minutes and lyophilized to afford GlA_20 _and GlA_40_, respectively. The same concentration of aqueous L-glutamine solution that was not autoclaved but filter sterilized served as control (GFs).

### Chemical characterization of autoclaved products

GlA_20_, GlA_40 _and control were subjected to TLC in order to study the structural changes in L-glutamine due to thermal degradation by autoclaving. Samples spotted on TLC plates were run in solvent systems comprising of butanol:water:acetic acid:: 4:1:5 and n-propanol:2-propanol:water:: 4:3:2 followed by heating at 300°C in an oven for 15 minutes. Visual changes in color and R_f _values of the spots were noted after spraying ninhydrin reagent on chromatographed TLC plate.

The autoclaved samples (GlA_20 _and GlA_40_) were also characterized by modern spectroscopic techniques i.e. NMR and ESI-MS. NMR spectra were recorded on a Bruker Avance-300 spectrometer. Mass spectra were recorded on QTOF-Micro of Waters Micromass.

### *Agrobacterium *growth in response to autoclaved products

The engineered *Agrobacterium tumefaciens *strain, GV3101 containing the plasmid p35SGUSINT with the *gus *reporter and *npt*II selection marker genes was used. To fresh cultures of this strain harvested at an optical density (OD) of 0.6 at A_600 _nm, 2.0 g litre^-1 ^of GlA_20_, GlA_40 _and GFs (as standardized in an earlier study) were added and grown overnight as shake cultures in 20 ml Yeast Mannitol Broth (YMB) in dark at 28°C and 150 rpm for 24 hrs. In another experiment, freshly revived cultures of *A. tumefaciens *was grown in liquid basal MS medium [17] containing either of 2.0 g l^-1 ^GFs, GlA_20_, GlA_40 _maintained at different pH i.e., 5.2, 5.6, 5.9 and 7.0. Growth in response to 2.0 g l^-1 ^5-oxo proline (purchased from Sigma, USA) in MS and YMB was also tested at these pHs. All the results were confirmed using 1.5% agar solidified YMB and MS medium.

For each of these experiments, cultures grown in medium free of GFs, GlA_20_, GlA_40 _or 5-oxo proline served as control. A minimum of three replicates per treatment were used. Growth in each case as represented by population density (i.e., a value = optical density (OD) at A_600 _nm × 1 × 10^9 ^cfu ml^-1^) was measured at regular 12 hr interval for 24 and 48 hrs of incubation in case of liquid and solid media, respectively.

### *Agrobacterium *virulence (*vir*) gene induction assay

*Vir *gene induction by the autoclaved products was tested using an octopine-type *Agrobacterium *strain A348 harboring the pSM219 plasmid with *lacZ *under the control of *virH *promoter in *trans *to the wild-type pTiA6 plasmid [18,19]. GlA_20_, GlA_40 _or GFs were added to overnight grown bacterial cultures in YMB. The reporter *β*-galactosidase activity was measured, and the results were expressed in specific units calculated as described [18-20]. The popular *vir *gene inducer AS (100 *μ*M) was used as a positive control for its maximal *vir *gene induction ability.

### Facilitation of *Agrobacterium *infection of plants by the autoclaved products of L-glutamine

The effect of GlA_20_, GlA_40 _or GFs on *Agrobacterium *infection of plants was tested using tender leaves of a number of plant species like *Podophyllum hexandrum *(Indian may apple), *Aloe vera *(aloe)*, Lavendula officinalis *(lavender), *Rosa *sp. (wild rose), *Malus domestica *(apple) rootstock MM106, *Dendrocalamus asper *(bamboo), *Cynodon dactylon *(grass), *Zea mays *(maize), *Oryza sativa (rice), Triticum aestivum (wheat), Aurocaria *(ornamental gymnosperm) and *Dryopteris *(fern). The leaves of *Nicotiana tabacum *(tobacco) served as control.

All the leaves were washed with Tween 20 and surface sterilized using 0.01% mercuric chloride for 5-10 min followed by thorough rinsing in sterile de-ionized water to remove all traces of mercuric chloride. The surface sterilized leaves were immersed for 10 min in fresh overnight grown culture of *A. tumefaciens *strain GV3101 and co-cultivated for 1, 2, 3, 5, 6 and 8 days at 28°C in dark after blotting off excess bacteria. Basal MS medium containing 2.0 g l^-1 ^of either of GlA_40_, GlA_20_, 5-oxoproline and GFs was used for co-cultivation. After each co-cultivation period, cefotaxime at 1 g l^-1 ^was used to wash the explants free of all residual *Agrobacterium* which were cultured on basal MS medium containing 5 μM TDZ and 10 μM NAA (MSC) at pH 5.6 under culture lab conditions for callus formation. In order to identify the optimal conditions, different pH, i.e., 5.2, 5.6, 5.9 and 7.0 of the co-cultivation medium containing 2.0 g l^-1 ^of GlA_40 _were also tested.

In another experiment, leaves of different plant species (tobacco, Indian may apple, aloe, lavender, wild rose, apple rootstock MM106, bamboo, grass, maize, rice, aurocaria and fern) were also transformed using different densities of *Agrobacterium *equivalent to 1 × 10^7^, 1 × 10^8^, 1 × 10^9 ^and 1 × 10^10 ^cfu ml^-1 ^at A_600 _nm. These were then co-cultivated on medium supplemented with either of GFs, GlA_40_, GlA_20 _or 5-oxo proline at pH 5.6.

*Agrobacterium *infected explants co-cultivated on medium containing 100 μM AS but free of GFs, GlA_40_, GlA_20 _or 5-oxo proline supplements served as positive control. A minimum of five replicates with three leaves per plant species were taken for each experiment which were repeated at least thrice.

### GUS assay

The histo-chemical assay of Jefferson *et al. *[21] was used to assess the success of transgene delivery into explants. After 1, 2, 3, 5, 6 and 8 days of co-cultivation, explants, treated (co-cultivated on media containing GFs, GlA_40_, GlA_20 _or 5-oxo proline at different pH) and control (co-cultivated on media containing 100 μM AS but free of either GlA_20_, GlA_40_, GFs or 5-oxo proline) were randomly selected and immersed in assay buffer containing 5-bromo-4-chloro-3-indolyl-ß-D-glucuronide (GUS) followed by vacuum infiltration for 15 minutes. After an overnight incubation in dark at 37°C, the expression of *gus *reporter gene was scored as blue spots and/or sectors per leaf explant and photographed using a Sony Cybershot DSC-F-828 camera. GUS assay was also done for the leaf explants transformed using different densities of *Agrobacterium *followed by co-cultivation on medium containing GFs, GlA_40_, GlA_20 _or 5-oxo proline as well as control.

### PCR confirmation of genetic transformation

Callus tissue formed on the leaf explants of different plant species transformed in the presence and absence of either GFs, GlA_40_, GlA_20 _or 5-oxo proline were selected on MSC containing 100 μg ml^-1 ^kanamycin. As described by Doyle and Doyle [22], total genomic DNA was extracted from the kanamycin resistant calli (500 mg). These were PCR amplified using 35 cycles of denaturation at 94°C for 1 min, annealing at 55°C for 1 min and extension at 72°C for 2 min followed by further extension cycle of 7 min at 72°C using a programmable Stratagene Robocycler Gradient 40. The isolated genomic DNA (50 ng) was amplified using 200 *μ*M dNTPs, 1.5 U Taq DNA polymerase and 10 pmol of forward and reverse primers i.e., 5'-GGTGGGAAAGCGCGTTACAAG-3' and 5'-TGGATCCCGGCATAGTTAAA-3', respectively (Bangalore Genei, India) designed so as to amplify a 490 bp fragment of the *gus *gene). While 50 pg of plasmid DNA served as positive control, DNA from untransformed tissues were used as negative controls. The amplified products were finally resolved on 1.2% agarose gel using a 0'GeneRuler™ 100 bp plus DNA ladder from Fermentas, Life Sciences. Plant species (eg. *Podophyllum *and *Aurocaria*) that failed to produce leaf callus were not subjected to PCR.

### Southern hybridization

Genomic DNA was isolated from callus tissues of different plant species transformed in presence and absence of α-amino glutarimide. The isolated DNA (10 μg) were digested with *Hind*III and *EcoR*I (New England Biolabs Inc. USA) and resolved on 0.8% agarose gel along with a 0'GeneRuler™ 100 bp plus DNA ladder (Fermentas, Life Sciences). This was then blotted onto a nylon membrane (Hybond-N, Amersham Biosciences, Little Chalfont, Buckinghamshire, UK) and hybridized with PCR amplified plasmid *gus *gene probe labeled with Biotin using Biotin DecaLabel DNA Labeling kit, Fermentas, Life Sciences. The chromogenic substrate BCIP/NBT (nitro blue tetrazolium chloride/5-bromo-4-chloro-3-indolyl phosphate, toluidine salt) was used to detect the hybridization products of the biotinylated probe and the streptavidin-alkaline phosphatase conjugate as blue-purple colored bands.

## Results

### Chemical characterization of autoclaved products

The yield of GlA_20 _and GlA_40 _was found in the range of 92-95%. While GlA_20 _was observed in white crystalline powder, GlA_40 _was a creamy white powder. The latter was highly hygroscopic and turned into a pale yellow sticky mass within an hour under ambient conditions.

### Thin Layer Chromatography

TLC analysis of GlA_20_, GlA_40 _and GFs showed distinct differences in their R_f _values. GlA_20 _showed only one spot at R_f _0.73, hence, confirmed the formation of only one product (II). However, in case of GlA_40_, one major spot was observed at R_f _0.70 together with a minor spot at R_f _0.73. The observation suggested the presence of one major compound (III) along with a minor compound (II) in GlA_40_. The difference in the R_f _values of both products from GFs (R_f _0.77) indicated the transformation of L-glutamine upon autoclaving.

### NMR and mass spectroscopy

**Figure 1:**
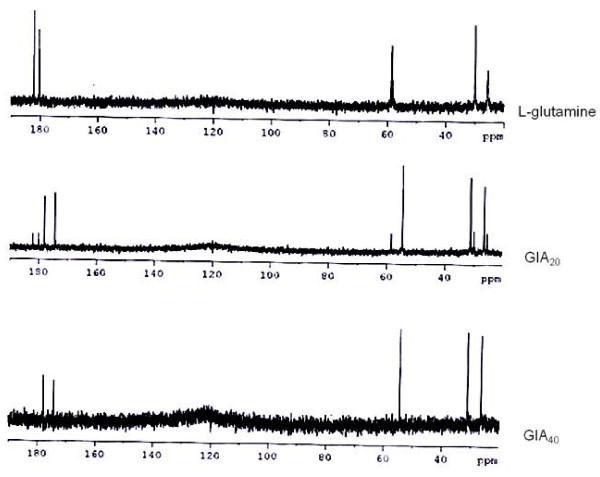
**NMR spectral data (^1^H, ^13^C and 2D experiments) of L-glutamine and its autoclaved products (GlA_20 _and GlA_40_)**.

The NMR spectral data (^1^H, ^13^C and 2D experiments) provided evidence for GlA_20 _and GlA_40 _being two structurally different compounds (Figure 1).

GlA_20 _was identified as 5-oxo proline (pyroglutamic acid, II) on the basis of comparison of NMR and mass spectral data with reported values. Electrospray ionization mass spectrometry (ESI-MS) of GlA_20 _showed protonated molecular ion at *m/z *130 [M+H]^+ ^which corresponded to the molecular formula C_5_H_7_NO_3_.

**Figure 2:**
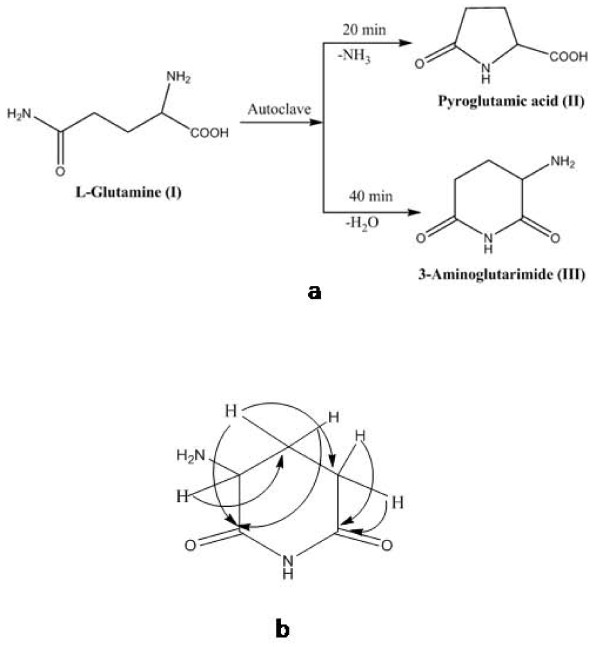
**(a) Chemical transformation of L-glutamine into pyroglutamic acid and 3- or α-amino glutarimide upon autoclaving at 121°C and 15 psi (b) Key HMBC of α-amino glutarimide**.

^13^C NMR spectrum of GlA_40 _was dominant with the signals of major product (III) that showed 5 carbon resonances, of which two are observed at δ 182.1 and 180.0 for carbonyls. A downfield shift of these resonances when compared with parent compound (control) indicated presence of a cyclic ring. The other 3 carbons resonated at δ 58.2, 29.9 and 25.5. Considerable shifts in these resonances in comparison to the respective resonances of control (L-glutamine) provided further support for the cyclic structure for product III. DEPT 135 experiment revealed the nature of these three resonances as two CH_2 _at δ 29.9 and 25.5 and one as CH at δ 58.2. In ^1^H NMR spectrum, three multiplets were observed at δ 1.93, 2.38 and 4.08, each integrating for two, two and one proton, respectively. The multiplet patterns of these three proton resonances due to mutual scalar couplings indicate that they are connected in series. The coupling pattern was further confirmed by COSY. Electrospray ionization mass spectrometry (ESI-MS) of GlA_40 _showed protonated molecular ion at *m/z *129 [M+H]^+ ^for major product (III) which corresponded to the molecular formula C_5_H_8_N_2_O_2_. The structural elucidation of compound III finally revealed complete cyclization of glutamine during autoclaving. HMQC and HMBC study further confirmed the loss of a molecule of water to form compound III i.e. α-amino glutarimide or 3-amino-2,6-piperidinedione (Figure 2a and 2b).

The results clearly suggested that the formation of α-amino glutarimide (III) was preferred over pyroglutamic acid (II) when L-glutamine was over autoclaved.

**α-amino glutarimide: **^1^H NMR δ: 1.93 (m, 2H), 2.38 (m, 2H), 4.08 (q, 1H); ^13^C NMR δ: 182.1, 180.0, 58.2, 29.9, 25.5; ESI-MS *m/z*: 129 [M+H]^+^.

### *Agrobacterium *growth in response to autoclaved products of L-glutamine

**Figure 3:**
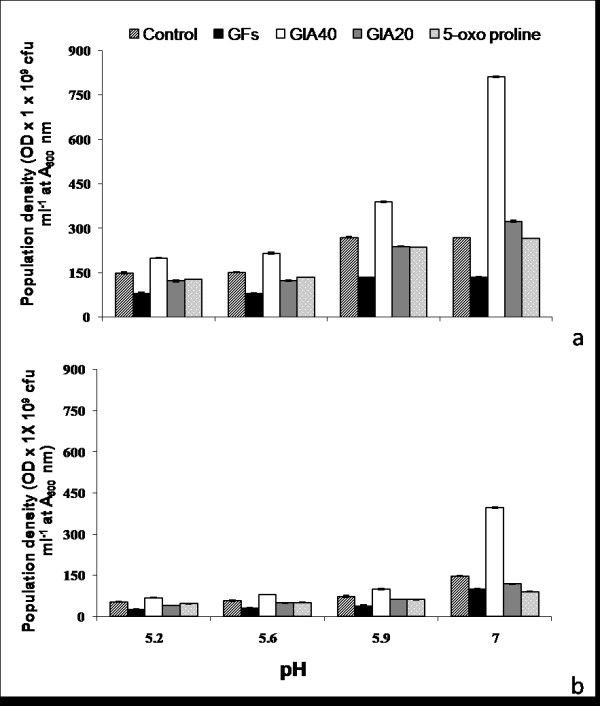
***A. tumefaciens *growth in (a) YMB and (b) MS as represented by OD × 1 × 10^9 ^cfu ml^-1 ^at A_600 _nm in response to 2.0 g l^-1 ^GFs, GlA_20_, GlA_40 _and control**.

**Figure 4:**
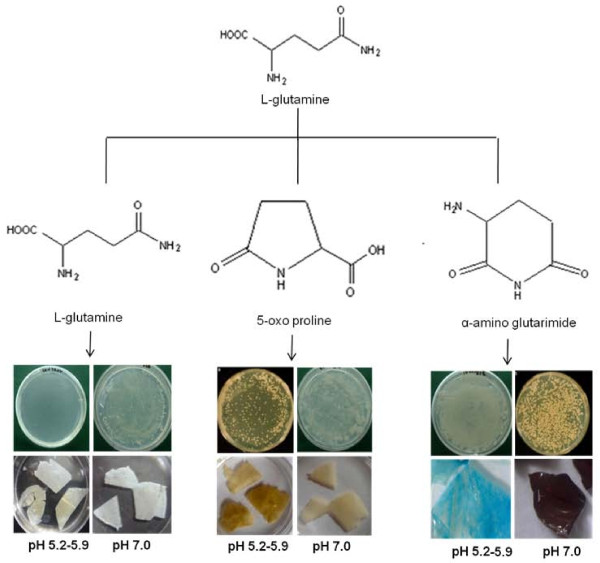
**Effect of pH (5.2-5.9 and 7.0) on *A. tumefaciens *growth and GUS expression of explants transformed in presence of GFs, GlA_20 _and GlA_40_**.

*A. tumefaciens *growth was more pronounced at pH 7.0 as compared to acidic pH (5.2 to 5.9), irrespective of supplements (GFs, GlA_20_, 5-oxo proline or GlA_40_,), and a similar trend was recorded in both YMB and MS (Figure 3). However, growth was remarkably higher in YMB as compared to MS. As compared to control, maximum growth was recorded at pH 7.0 in the presence of GlA_40 _(i.e, 3.0 and 2.68 fold in YMB and MS, respectively). While growth in the presence of GlA_20 _was 1.2 and 0.8 fold, that in the presence of 5-oxo proline was about 1.0 and 0.6 fold in YMB and MS, respectively. Growth in the presence of GFs was always lower than control, irrespective of culture media. At acidic pH of YMB ranging from 5.2 to 5.6, growth was lower than that in the presence of GFs (0.5 to 0.54 fold), GlA_20 _(0.81 to 0.89 fold) and 5-oxo proline (0.88 fold). However, about 1.4 fold growth was recorded in the presence of GlA_40 _(Figures 3 and 4). In case of MS, a trend similar to that in YMB was observed. While growth was lower by 0.5 fold in the presence of GFs, it ranged between 0.75 to 0.88 fold in the presence of GlA_20 _and 5-oxo proline. However, growth in presence of GlA_40 _was 1.3 to 1.4 fold.

### β-galactosidase activity as a measure of *Agrobacterium *virulence (*vir*) gene induction

Considerably high β-galactosidase activity was recorded in the presence of AS, a known *vir *gene inducer. However, the activity of control (no additions) was lower than that in the presence of AS (Table 1). While the activities were 132.7 and 132.0 Miller units in the presence of GFs and GlA_20_, respectively, suppression (130.9 Miller units) was observed in the presence of GlA_40_.

**Table 1:** *vir *gene induction by L-glutamine (GFs) and its autoclaved products (GlA_20 _and GlA_40_)

Compound tested	β-galactosidase activity ( Miller units)
Control	58.9 ± 0.6

AS (200 μM)	148.0 ± 1.1

GFs	132.7 ± 0.7

GlA_20_	132.0 ± 0.6

GlA_40_	130.9 ± 0.9

### *Agrobacterium *infection of resistant host plants by autoclaved products of L-glutamine

*A. tumefaciens *growth on leaf explants varied with plant species when transformed with different cell densities and co-cultivated on MS supplemented with GlA_40_. No growth was observed on any of the explants when transformed with 1 × 10^7 ^cfu ml^-1 ^of *A. tumefaciens *followed by co-cultivation on MS containing either of GFs, GlA_20 _or 5-oxo proline. Slight *A. tumefaciens *growth was however, recorded on explants of bamboo, maize and apple rootstock transformed using a cell density of 1 × 10^7 ^cfu ml^-1 ^followed by co-cultivation in the presence of GlA_40_. No explant, except tobacco showed *A. tumefaciens *growth when co-cultivated on control medium after transformation using cell densities up to 1 × 10^8 ^cfu ml^-1. ^However, explants of apple rootstock, bamboo, maize, and rice showed slight growth on control medium at all tested cell densities beyond 1 × 10^8 ^cfu ml^-1^.

**Figure 5:**
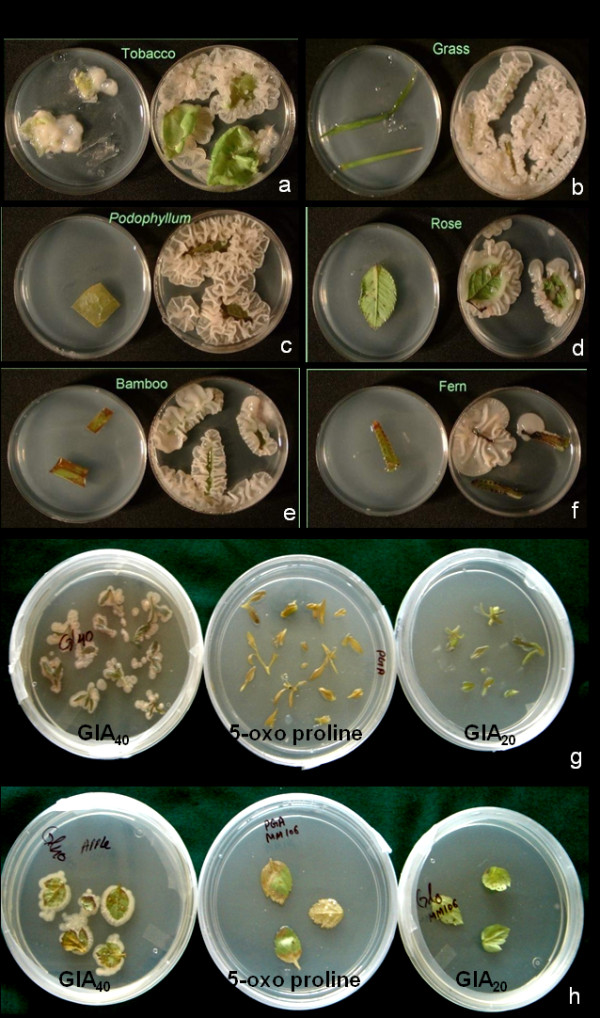
***Agrobacterium *****growth on leaf explantsof;** (a) tobacco, (b) grass, (c) Indian may apple, (d) rose, (e) bamboo and (f) fern (transformed using 1 × 10^9 ^cfu ml^-1 ^followed by co-cultivation on MS containing GlA_40 _at pH 5.6); (g-h) growth in response to GlA_40_, 5-oxo proline and GlA_20 _(g: lavender, h: apple rootstock MM106).

Growth increased with further increase in cell density i.e., lowest at 1 × 10^8 ^cfu ml^-1 ^and highest at 1 × 10^10 ^cfu ml^-1 ^in case of Indian may apple, aloe, lavender, wild rose, apple rootstock, bamboo, grass, maize and rice; and also in the leaf explants of fern and *Aurocaria *when co-cultivated on MS containing GlA_40 _(Figure 5). Even in case of control i.e., in the absence of supplements, *A. tumefaciens *growth was observed on apple rootstock and rice when they were transformed using either of 1 × 10^9 ^or 1 × 10^10 ^cfu ml^-1^. However, *A. tumefaciens *failed to grow on any of the explants when either GlA_20 _or 5-oxo proline was present in the co-cultivation medium, irrespective of cell densities, (Sigma, USA).

When pH of the co-cultivation medium was considered, depending upon the plant species, slight to profuse growth was recorded on or around the explants in the acidic pH range only. In contrast, no *A. tumefaciens *growth was recorded on or around the explant surface at pH 7.0, irrespective of supplements.

### GUS expression

GUS expression varied with *A. tumefaciens *density, plant species or presence or absence of GlA_40 _(Table 2). Irrespective of the presence or absence of GlA_40 _in the co-cultivation medium, tobacco showed GUS expression at all cell densities ranging from 1 ×10^7 ^and 1 × 10^9 ^cfu ml^-1^. On the other hand, the presence of GlA_20 _or 5-oxo proline failed to induce GUS expression in all the studied plant species including tobacco.

**Table 2:** GUS expression after 2 days of co-cultivation in leaf explants transformed using different *A. tumefaciens *population densities

S. No.	Plant species	*A. tumefaciens *population density (cfu ml^-1^) versus GUS expression in different plant species															
		
		**1 × 10**^**7**^				**1 × 10**^**8**^				**1 × 10**^**9**^				**1 × 10**^**10**^			
		
		Cont*	**GlA**_**20**_	5-oxo proline	**GlA**_**40**_	Cont	**GlA**_**20**_	5-oxo proline	**GlA**_**40**_	Cont	**GlA**_**20**_	5-oxo proline	**GlA**_**40**_	Cont	**GlA**_**20**_	5-oxo proline	**GlA**_**40**_
1.	*Nicotiana tabacum *(tobacco)	++	-	-	++	++	-	-	++	+++++	-	-	+++++	-	-	-	-

2.	*Podophyllum hexandrum *(Indian may apple)	-	-	-	-	-	-	-	+	-	-	-	++++	-	-	-	-

3.	*Aloe vera *(aloe)	-	-	-	-	-	-	-	+	-	-	-	++	-	-	-	++

4.	*Lavendula officinalis *(lavender)	-	-	-	-	-	-	-	+	-	-	-	+++	-	-	-	-

5.	*Rosa *sp. (Wild rose)	-	-	-	-	-	-	-	-	-	-	-	+++	-	-	-	-

6.	*Malus domestica *(apple rootstock)	-	-	-	+	-	-	-	++	-	-	-	+++++	-	-	-	-

7.	*Dendrocalamus asper *(bamboo)	-	-	-	++	-	-	-	+++	-	-	-	+++++	-	-	-	++++

8.	*Cynodon dactylon *(grass)	-	-	-	-	-	-	-	+	-	-	-	++++	-	-	-	++++

9.	*Zea mays *(maize)	-	-	-	+	-	-	-	++	-	-	-	+++	-	-	-	+++

10.	*Oryza sativa *(rice)	-	-	-	-	-	-	-	+++	+++	-	-	++++	+++	-	-	++++

11.	*Aurocaria *(gymnosperm)	-	-	-	-	-	-	-	-	-	-	-	++++	-	-	-	++++

12.	*Dryopteris *(fern, pteridophyte)	-	-	-	-	-	-	-	-	-	-	-	++++	-	-	-	++++

where Cont* = control; + = one to five tiny blue spots; ++ = one to two large blue spots; +++ = small or faint blue patches; ++++ = large blue patch covering almost quarter of the leaf surface; and +++++ = entire leaf showed distinct blue color

**Figure 6:**
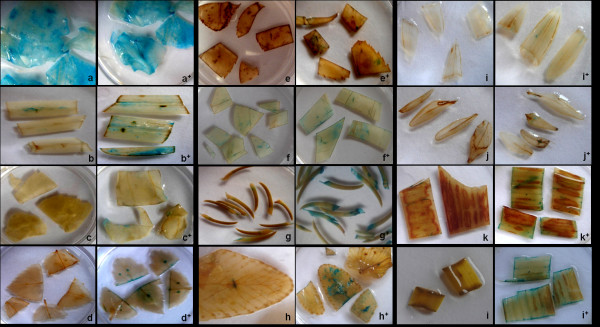
**GUS expression of leaf explants transformed using 1 × 10^9 ^cfu ml^-1 ^followed by co-cultivation at pH 5.6. **(a) tobacco, (b) grass (c) Indian may apple, (d) apple rootstock, (e) rose, (f) bamboo, (g) aurocaria (h) fern, (i) aloe, (j) lavender (k) maize and (l) rice (^+^denotes the presence of GlA_40_).

GUS expression improved with increase in *A. tumefaciens *cell densities from 1 × 10^7 ^to 1 × 10^9 ^cfu ml^-1 ^in the presence of GlA_40 _in case of apple rootstock, bamboo and maize; and from 1 × 10^8 ^to 1 × 10^9 ^cfu ml^-1 ^in Indian may apple, aloe, lavender, grass and rice. However, the best response in terms of strong GUS expression spread over a larger area was recorded, only when 1 × 10^9 ^cfu ml^-1 ^of *A. tumefaciens *was used, irrespective of plant species (Figure 6). Increase in cell density beyond 1 × 10^9 ^cfu ml^-1 ^had no effect on GUS expression (or in other words transformation) in case of aloe, grass, maize, rice, aurocaria and fern. On the other hand, with time, the explants turned necrotic due to *A. tumefaciens *overgrowth at 1 × 10^10 ^cfu ml^-1 ^in case of tobacco, Indian may apple, lavender, wild rose and apple rootstock.

The leaf explants of all the studied plant species showed GUS expression only in the acidic pH range when co-cultivated on MSC containing GlA_40_. However, the pH optima and the intensity of expression varied with the plant species tested (Table 3). While the leaf explants of tobacco, lavender, apple rootstock, bamboo, maize and rice showed GUS expression at all pH in the acidic range (i.e., 5.2, 5.6 and 5.9), those of Indian may apple, wild rose and grass showed GUS expression at pH 5.6 and 5.9 only. On the other hand, the explants of aloe and fern tested positive when co-cultivated at pH 5.2 and 5.6, whereas, that in aurocaria was best observed at pH 5.6 only.

**Table 3:** GUS expression in response to different pH during co-cultivation on MSC containing GlA_40 _after transformation of leaf explants of different plant species using 1 ×10^9 ^cfu ml^-1 ^*A. tumefaciens*

S. No.	Plant Species	pH	GUS Expression	
			
			(+) GlA_40_	(-) GlA_40_
1.	*Nicotiana tabacum *(tobacco)	5.2	+++++ (< 1 day)	+++++ (< 1 day)
		
		5.6	+++++ (< 1 day)	+++++ (< 1 day)
		
		5.9	+++++ (< 1 day)	+++++ (< 1 day)
		
		7.0	-	-

2.	*Podophyllum hexandrum *(Indian may apple)	5.2	-	-
		
		5.6	++++ (2 days)	-
		
		5.9	+++ (2 days)	-
		
		7.0	-	-

3.	*Aloe vera *(aloe)	5.2	+++ (1 day)	-
		
		5.6	+++ (1 day)	-
		
		5.9	-	-
		
		7.0	-	-

4.	*Lavendula officinalis *(lavender)	5.2	+++ (1 day)	-
		
		5.6	++++ (1 day)	-
		
		5.9	+++ (1 day)	-
		
		7.0	-	-

5.	*Rosa *sp. (Wild rose)	5.2	-	-
		
		5.6	+++ (1 day)	+ (4 days)
		
		5.9	+++ (1 day)	+ (5 days)
		
		7.0	-	-

6.	*Malus domestica *(apple rootstock)	5.2	++++ (2 days)	+ (3 days)
		
		5.6	++++ (2 days)	+ (3 days)
		
		5.9	++++ (2 days)	+ (3 days)
		
		7.0	-	-

7.	*Dendrocalamus asper *(bamboo)	5.2	+ (2 days)	+ (6 days)
		
		5.6	+++ (1 day)	+ (3 days)
		
		5.9	++ (1 day)	+ (4 days)
		
		7.0	-	-

8.	*Cynodon dactylon* (grass)	5.2	-	-
		
		5.6	++ (1 day)	+ (5 days)
		
		5.9	++ (1 day)	+ (3 days)
		
		7.0	-	-

9.	*Zea mays *(maize)	5.2	+++ (1 day)	+ (3 days)
		
		5.6	+++ (1 day)	+ (3 days)
		
		5.9	++++ (1 day)	+ (5 days)
		
		7.0	-	-

10.	*Oryza sativa *(rice)	5.2	++ (1 day)	+ (4 days)
		
		5.6	+++ (1 day)	+ (2 days)
		
		5.9	+++ (1 day)	+ (2 days)
		
		7.0	-	-

11.	*Aurocaria *(gymnosperm)	5.2	-	-
		
		5.6	++++ (3 days)	-
		
		5.9	-	-
		
		7.0	-	-

12.	*Dryopteris *(fern, pteridophyte)	5.2	++ (1 day)	+ (7 days)
		
		5.6	+++ (1 day)	+ (3 days)
		
		5.9	-	-
		
		7.0	-	-

where + = one to five tiny blue spots; ++ = one to two large blue spots; +++ = small or faint blue patches; ++++ = large blue patch covering almost quarter of the leaf surface; +++++ = entire leaf showed distinct blue color and - = no GUS expression (Values in parenthesis represent the duration of co-cultivation)

In case of media containing 100 μM AS, but free of all glutamine supplements, longer co-cultivation time was required for the explants of wild rose, apple rootstock, bamboo, grass, maize, rice and fern (Table 3). The only exception was tobacco where < 1 day of co-cultivation was sufficient for strong GUS expression. On the other hand, no GUS expression was observed in explants of any of the plant species co-cultivated at pH 7.0 (Table 3; Figure 4).

### PCR confirmation of genetic transformation

**Figure 7:**
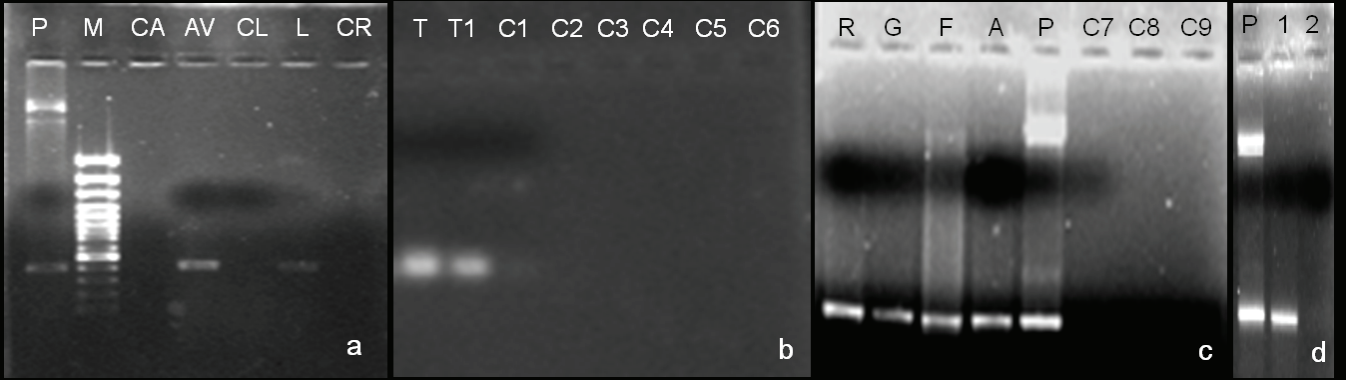
**PCR of genomic DNA from leaf callus showing amplification of 490 bp gus gene.** (a) Lane P: plasmid, Lane M: 0'GeneRuler™ 100 bp plus DNA ladder (Fermentas, Life Sciences), Lanes CA-AV: transformed aloe (CA: in absence of GlA_40 _and AV: in presence of GlA_40_), Lanes CL and L: transformed lavender (CL: in absence of GlA_40_, and L: in presence of GlA_40_), Lane CR: transformants in absence of GlA_40 _(CR: wild rose); (b) Lane T-T1: transformed tobacco (T: in absence of GlA_40_, T1: in presence of GlA_40_), Lanes C1 to C6: untransformed controls (1: aloe, 2: lavender, 3: wild rose, 4: grass, 5: fern, 6: apple rootstock); (c) Lanes R, G, F, A: transformed calli in presence of GlA_40 _(R: wild rose, G: grass, F: fern, A: apple rootstock) P: plasmid and Lanes C7-C9: transformants in absence of GlA_40 _(7: apple rootstock, 8: fern, 9: grass) and (d) Lane P: Plasmid DNA, Lane 1: maize transformed in presence of GlA_40_; Lane 2: transformant in absence of GlA_40_.

PCR amplification products of about 490 bp corresponding to *gus *gene were observed in the leaf calli of aloe, lavender, tobacco, rose, grass, fern and apple rootstock and maize provided GlA_40 _was present in the co-cultivation medium (Figure 7a, b, c and 7d). However, amplification was not detected in the calli derived from explants co-cultivated on either control or on media containing GFs, GlA_20 _or AS.

### Southern hybridization

**Figure 8:**
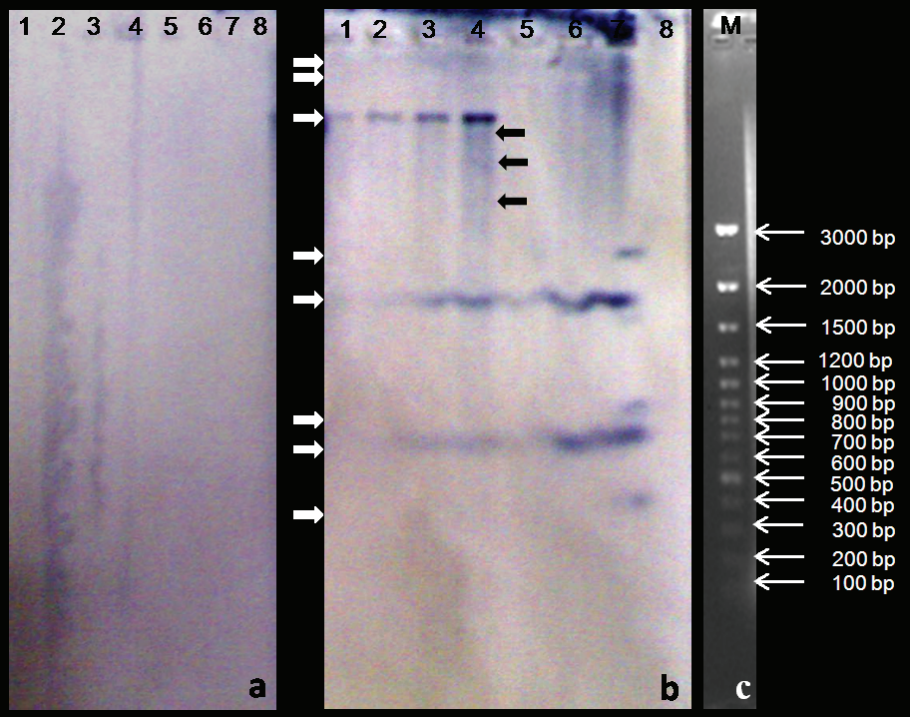
**Southern blot of genomic DNA from leaf callus hybridized with Biotin labeled *gus *gene probe.** (a) Control Lanes 1-5: transformants in absence of GlA_40 _but presence of AS (1 and 2: aloe, 3: wild rose, 4: maize, 5: fern, 6: lavender, 7: grass (b) transformants in presence of GlA_40 _(Lane 1 and 2: aloe, 3: wild rose, 4: maize, 5: fern, 6 and 7: lavender, 8: grass); (c ) M: 0'GeneRuler™ 100 bp plus DNA ladder (Fermentas, Life Sciences).

Genomic DNA of calli derived from aloe, wild rose, maize, fern, lavender and grass leaves transformed using *A. tumefaciens *density of 1 × 10^9 ^cfu ml^-1 ^and co-cultivated in the presence of GlA_40 _tested positive in Southern hybridization and distinct purple-blue signals were detected (Figure 8). However, no hybridization signals were observed in case of leaf calli obtained from explants co-cultivated in the presence of AS but in absence of GlA_40 _(Figure 8a). Hybridization signals were also not detected in the untransformed leaf calli of the studied plant species (not shown). Only tobacco and apple rootstock MM106 showed the hybridization signals both in the presence or absence of GlA_40 _in the co-cultivation medium (not shown). A distinct single band (> 3 kb) was observed in case of aloe (Figure 8b, lanes 1 and 2). While a distinctly sharp band of > 3 kb was observed along with > 1.5 kb and 700 bp bands in wild rose (Figure 8b, lane 3), four bands above 3 kb and two bands of about 1.8 kb and 700 bp were observed in maize (Figure 8b, lane 4). No signal was observed in case of fern (Figure 8b, lane 5) whereas, two sharp bands of about 1.8 kb and 700 bp were observed in case of lavender (Figure 8, lanes 6 and 7). Three distinct bands (400 bp, 900 bp and 2.8 kb) were observed in case of grass (Figure 8, lane 8).

## Discussion

L-glutamine is a highly thermolabile and non-volatile biomolecule that is chemically modified into different compounds at high temperatures. When present in aqueous solution at pH values that are not extreme, L-glutamine is transformed in two step reactions, first into glutamic acid and then into pyroglutamic acid or 5-pyrrolidone-2-carboxylic acid or 5-oxo proline and ammonia [23]. Besides 5-pyrrolidone-2-carboxylic acid [24], L-glutamine undergoes pyrolysis at temperatures as high as 300°C leading to the formation of nitrogen containing heterocycles [25]. The additional single methylene group in the side chain allows the free form of glutamine to cyclize and de-amidate spontaneously into pyrrolidone carboxylic acid, a five-membered ring structure found at the N-terminus of many immunoglobulin polypeptides [26]. In the present study also, L-glutamine underwent cyclization to form 5-oxoproline and α-amino glutarimide. However, this occurred when L-glutamine was autoclaved at 121°C and 15 psi for 20 and 40 min, respectively (Figure 2a and 2b). Further chemical investigations using TLC and different spectroscopic methods revealed that irrespective of the duration of autoclaving, both the compounds could be detected in the same sample. However, the amount of α-amino glutarimide was more (92-95%) when the aqueous solution of L-glutamine was autoclaved for 40 min. This indicated the possibility of easy synthesis of α-amino glutarimide simply by autoclaving aqueous solutions of L-glutamine for 40 min. It would be pertinent to mention that in general, the synthesis of the glutarimide ring requires a minimum of 2-3 step reactions [8,27].

**Figure 9:**
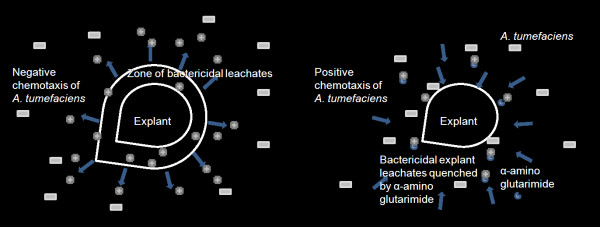
**A hypothetical diagram showing how α-amino glutarimide facilitates *A. tumefaciens *infection of non-host explants that exude bactericidal/bacteriostatic leachates**.

Glutarimides are extremely valuable biomolecules that act as a carrier or vector for transporting biologically active functional groups through cell membranes. The structural features and physiochemical properties of the glutarimide moiety is remarkably similar to the uracil derivatives. Therefore, it interacts with specific receptors involved in transport of uracil and thymine nucleosides across biological membranes [28,29]. Being a constituent of a large number of other important compounds, the glutarimide moeity possesses a broad spectrum of pharmacological and anticancer properties [2,30,31]. Particularly, 2,6-piperidinedione, a glutarimide with an intact imide group (OC-NH-CO) substituted at α and β position in the ring acts as a powerful inhibitor of human rhinovirus 3C protease [32]. However, in the present study α-amino glutarimide was found to have a new function of facilitating *Agrobacterium *infection of different susceptible and resistant plant species that ranged from host to non-hosts (Tables 2 and 3; Figures 3, 4, 5, 6, 7 and 8). The present study also found that neither 5-oxoproline nor 3-amino glutarmide are inducers of the *vir *regulon (Table 1). Rather, the compound promoted *A. tumefaciens *growth resulting in an increase in population density. However, increase in population density beyond 1 × 10^9 ^cfu ml^-1 ^was found to have a detrimental effect on transformation efficiency (Table 2). This led us to hypothesize that α-amino glutarimide formed due to autoclaving of L-glutamine was probably responsible for the xenobiotic quenching of the toxic wound exudates from resistant explants as has been indicated in tea [33]. This appears to be so because most of the plant species that were tested in the present study, leached exudates that not only killed the studied explants (data not shown) but also retarded *A. tumefaciens *growth as has been shown in tea in our earlier study [33]. These bactericidal leachates probably killed or prevented the chemotaxis of an optimal *A. tumefaciens *population density required for explant infection. Not surprisingly, the possibility of a strong negative influence by the toxic polyphenolic exudates on the chemotactic movement of *Agrobacteria *towards the explant has been considered [34]. On the other hand, when the bactericidal-explant-exudates were possibly quenched by α-amino glutarimide, the requisite population density of *A. tumefaciens *which would otherwise have been repelled or killed, were now allowed to move towards the explants for infection (Figure 9a and 9b).

However, the activity of GlA_40 _was found to be pH dependant. While *Agrobacterium *growth was enhanced at pH 7.0, infectivity of explants of many of the studied plant species was mostly enhanced at pH 5.6 and some at pH 5.2 and 5.9, but never at pH 7.0 (Table 3 and Figure 4). This indicated the necessity of manipulation of pH of the media containing this compound for its appropriate utilization. Actually, *vir *gene induction and consequent *Agrobacterium *infection of plants is best facilitated at acidic pH [35]. However, the pH optima are known to vary with plant species for *Agrobacterium *virulence and gene transfer [36-38]. Designing adequate artificial environment and manipulation of wound responses of plant tissues has facilitated successful genetic transformation of many plant species by favoring plant-*A. tumefaciens *interactions.

## Conclusions

5-oxo proline and α-amino glutarimide were synthesized from L-glutamine upon autoclaving at 121°C and 15 psi for 20 or 40 min. Although the compound did not have any *vir *gene inducing property, α-amino glutarimide facilitated *Agrobacterium *infection of a number of resistant to susceptible plants successfully.

## Authors' contributions

IS and AB conceptualized and designed the study with valuable inputs from AG, JK and PSA. IS also performed the bench work on *Agrobacterium *mediated transformation of a few representative recalcitrant plants using 3-amino glutarimide. AB drafted the manuscript. US validated the findings and conceptualized the mechanism of action of 3-amino glutarimide in *Agrobacterium *mediated transformation. DK carried out the Southern hybridization, SS carried out the GUS assays for the different plants and JD carried out the β-galactosidase assays. AG participated in the conceptualization of the chemical studies. JK identified and characterized the compound formed due to autoclaving at different time intervals, NK validated the synthesis of 3-amino glutarimide through autoclaving while PD and BS participated in the validation studies. PSA convened and participated in the overall study. He also provided critical comments. All authors read and approved the final manuscript.
